# Prognosis prediction model for conversion from mild cognitive impairment to Alzheimer’s disease created by integrative analysis of multi-omics data

**DOI:** 10.1186/s13195-020-00716-0

**Published:** 2020-11-10

**Authors:** Daichi Shigemizu, Shintaro Akiyama, Sayuri Higaki, Taiki Sugimoto, Takashi Sakurai, Keith A. Boroevich, Alok Sharma, Tatsuhiko Tsunoda, Takahiro Ochiya, Shumpei Niida, Kouichi Ozaki

**Affiliations:** 1 Medical Genome Center, National Center for Geriatrics and Gerontology, Obu, Aichi Japan; 2 Department of Medical Science Mathematics, Medical Research Institute, Tokyo Medical and Dental University (TMDU), Tokyo, Japan; 3 RIKEN Center for Integrative Medical Sciences, Yokohama, Kanagawa Japan; 4 The Center for Comprehensive Care and Research on Memory Disorders, National Center for Geriatrics and Gerontology, Obu, Aichi Japan; 5 Department of Cognitive and Behavioral Science, Nagoya University Graduate School of Medicine, Nagoya, Aichi Japan; 6 Institute for Integrated and Intelligent Systems, Griffith University, Brisbane, Australia; 7 University of the South Pacific, Suva, Fiji; 8 Department of Biological Sciences, Graduate School of Science, The University of Tokyo, Tokyo, Japan; 9 Division of Molecular and Cellular Medicine, Fundamental Innovative Oncology Core Center, National Cancer Center Research Institute, Tokyo, Japan; 10 Institute of Medical Science, Tokyo Medical University, Tokyo, Japan

**Keywords:** Alzheimer’s disease, Biomarkers for early diagnosis, eQTL effect

## Abstract

**Background:**

Mild cognitive impairment (MCI) is a precursor to Alzheimer’s disease (AD), but not all MCI patients develop AD. Biomarkers for early detection of individuals at high risk for MCI-to-AD conversion are urgently required.

**Methods:**

We used blood-based microRNA expression profiles and genomic data of 197 Japanese MCI patients to construct a prognosis prediction model based on a Cox proportional hazard model. We examined the biological significance of our findings with single nucleotide polymorphism-microRNA pairs (miR-eQTLs) by focusing on the target genes of the miRNAs. We investigated functional modules from the target genes with the occurrence of hub genes though a large-scale protein-protein interaction network analysis. We further examined the expression of the genes in 610 blood samples (271 ADs, 248 MCIs, and 91 cognitively normal elderly subjects [CNs]).

**Results:**

The final prediction model, composed of 24 miR-eQTLs and three clinical factors (age, sex, and *APOE4* alleles), successfully classified MCI patients into low and high risk of MCI-to-AD conversion (log-rank test *P* = 3.44 × 10^−4^ and achieved a concordance index of 0.702 on an independent test set. Four important hub genes associated with AD pathogenesis (*SHC1*, *FOXO1*, *GSK3B*, and *PTEN*) were identified in a network-based meta-analysis of miR-eQTL target genes. RNA-seq data from 610 blood samples showed statistically significant differences in *PTEN* expression between MCI and AD and in *SHC1* expression between CN and AD (*PTEN*, *P* = 0.023; *SHC1*, *P* = 0.049).

**Conclusions:**

Our proposed model was demonstrated to be effective in MCI-to-AD conversion prediction. A network-based meta-analysis of miR-eQTL target genes identified important hub genes associated with AD pathogenesis. Accurate prediction of MCI-to-AD conversion would enable earlier intervention for MCI patients at high risk, potentially reducing conversion to AD.

**Supplementary information:**

**Supplementary information** accompanies this paper at 10.1186/s13195-020-00716-0.

## Background

Mild cognitive impairment (MCI) is an intermediate stage between normal aging and dementia, and its presence is associated with a higher risk of progression to clinically probable Alzheimer’s disease (AD) [1–3]. The annual conversion rate from MCI to AD has been reported as 10 to 15% [4]. After 6 years of follow-up, approximately 80% of MCI patients will have converted to AD (MCI converters [MCI-C]) [4, 5], although some MCI patients remain stable or convert back to normal (MCI non-converters [MCI-NC]) [6]. To date, there are no curative treatments for patients who already have AD, and available treatments are only able to postpone the progression of the disease [7]. Therefore, biomarkers for early detection of MCI-C and prognosis prediction models are both desperately required. These will allow early treatment of patients with MCI before they convert to AD, which could reduce the number of patients with AD.

Multifactorial diseases, such as AD, are induced by a combination of genetic and environmental factors. The heritability of late-onset AD is estimated to be around 60–80% [8]. Therefore, many genetic factors will undoubtedly contribute to the etiopathogenesis and progression of AD. In fact, a large number of genetic factors have already been associated with increased risk for AD. Amyloid precursor protein (*APP*), presenilin 1 (*PSEN1*), and presenilin 2 (*PSEN2*) as causes of autosomal dominant AD [9] and the ε4 allele of apolipoprotein E (*APOE* ε4) are the strongest known genetic risk factors [10, 11]. Some additional genetic loci (single nucleotide polymorphisms [SNPs]) associated with AD diagnosis have been recently identified by genome-wide association studies [12–14]. However, a large proportion of the heritability remains unexplained, and further investigation of novel genetic factors will be necessary for early detection of MCI-C.

As with genetic factors, microRNAs (miRNAs) have been examined as potential biomarkers for early AD prediction [15, 16]. MicroRNAs are small non-coding RNA molecules of approximately 22 nucleotides that play a key role in the post-transcriptional regulation of gene expression and cell development, including neural cells [17]. Changes in the expression of some miRNAs have been detected in neurons of patients with AD and other neurodegenerative diseases [18–22]. Also, neurite and synapse destruction associated with the pathology of neurodegenerative diseases has been detected in vitro by quantitative analysis of brain-enriched cell-free miRNA in human blood [23]. These miRNAs may have practical clinical use as blood-based biomarkers, which are attractive because they are minimally invasive, cost-effective, and easy to reproduce.

A powerful and useful method to investigate the relationship between SNPs and miRNA expression is to analyze the genome-wide expression quantitative trait loci (miR-eQTL) [24]. Some miR-eQTLs have been associated with disease pathogenesis, such as the pathogenesis of autoimmune disease [25], cancers [26], and neurological disorders [27], indicating that miR-eQTLs can be effective biomarkers for predicting disease prognosis.

Here, we examined comprehensive miRNA expression profiles and genetic variants in 197 MCI patients (83 MCI-C and 114 MCI-NC) and identified miR-eQTLs that could allow earlier diagnosis and therapeutic intervention. Our final prognosis prediction model was constructed based on a Cox proportional hazard model that included 24 miR-eQTLs and three clinical factors (age, sex, and *APOE* ε4 status). In an independent test set, the model successfully classified the MCI patients into groups at high and low risk of MCI-to-AD conversion. In addition, a network-based meta-analysis using miRNA target genes revealed important hub genes associated with the pathogenesis of AD. Statistically significant differences in expression between disease groups were observed for some of these hub genes. Accurate prediction of MCI-to-AD conversion risk could enable earlier interventions for appropriate patients, potentially leading to a reduction in conversions from MCI to AD.

## Methods

### Clinical samples

All 197 MCI patients and the associated clinical data were distributed from the National Center for Geriatrics and Gerontology (NCGG) Biobank, which collects human biomaterials and data for geriatrics research. The AD and MCI subjects were diagnosed with probable or possible AD based on the criteria of the National Institute on Aging Alzheimer’s Association workgroups [1, 2]. We included patients with probable AD as subjects in this study. The diagnosis of all subjects was made based on medical history, physical examination, diagnostic tests, neurological examination, neuropsychological tests, and brain imaging with magnetic resonance imaging or computerized tomography by one or more experts in dementia who are familiar with its diagnostic criteria (neurologists, psychiatrists, geriatricians, or neurosurgeons). Comprehensive neuropsychological tests included the Mini-Mental State Examination (MMSE), Alzheimer’s Disease Assessment Scale Cognitive Component Japanese version, Logical Memory I and II from the Wechsler Memory Scale–Revised, frontal assessment battery, Raven’s colored progressive matrices, and the Geriatric Depression Scale [28]. For all subjects, the number of the *APOE*4 alleles (the major genetic risk factor for AD) and the MMSE score were obtained. All subjects were required to be over 60 years of age.

### SNP genotyping and data cleaning

All 197 subjects were genotyped using the Affymetrix “Japonica Array” (TOSHIBA, Inc.) [29]. Genotype imputation was performed using IMPUTE2 (version 2.3.2) [30] with the 1000 Genomes Project reference panel. A total of 2,836,104 autosomal SNPs and short indels passed the quality control criteria after imputation (SNP/indel call rate ≥ 0.99 and minor allele frequency ≥ 0.01). To generate a set of independent SNPs, we further performed linkage disequilibrium-based SNP pruning using the statistical analysis program PLINK, version 1.90b [31] with a window size of 50 SNPs, a step of 5 SNPs, and a pairwise *r*^2^ threshold of 0.1 (--indep-pairwise 50 5 0.1). Any sites with missing data were discarded. A final subset of 92,878 SNPs was obtained.

### miRNA expression

Total RNA was extracted from a blood sample by using the 3D-Gene RNA extraction reagent from a liquid sample kit (Toray Industries, Inc.). The miRNA expression analysis was performed with a 3D-Gene miRNA Labeling kit and a 3D-Gene Human miRNA Oligo Chip (Toray Industries, Inc.), which was designed to detect 2562 miRNA sequences registered in miRBase release 21 (http://www.mirbase.org/). Normalization of miRNA expression was performed in the following steps. Mean and standard deviation (SD) were calculated by using a set of pre-selected negative control signals (background signals) from which the top and bottom 5% of values were removed. Signal values greater than mean + 2SD of the background signals were replaced with log2(signal − mean) and labeled effective signals. The remaining signal values were replaced with the minimum of the effective signals − 0.1. Undetected signal values were replaced by the minimum value of each miRNA. To normalize the signals across different microarrays, a set of pre-selected internal control miRNAs (miR-149-3p, miR-2861, and miR-4463) was used; these miRNAs had been stably detected in more than 500 serum samples. Each miRNA signal value was standardized by dividing it by the average signal of the three internal control miRNA signals [32].

### Construction of the prognosis prediction model

All data were strictly separated into a discovery cohort and a validation cohort. To generate datasets of pre-selected SNPs, we used two thirds of the entire discovery cohort to calculate *P* values in each cross-validation step. The *P* values corresponding to the SNPs were calculated with the following logistic regression model between MCI-C and MCI-NC:

$$ \mathrm{logit}(P)={\beta}_0+{\beta}_1\times \mathrm{age}+{\beta}_2\times \mathrm{sex}+{\beta}_3\times APOE4+{\beta}_4\times {X}_{\mathrm{SNP}}, $$

where *X*_SNP_ is the SNP genotypes and (*β*_1_, *β*_2_, …, *β*_*n*_) is the respective coefficients. The logistic regression was implemented using PLINK [31]. From *p* pre-selected SNPs (*p* = 100, 200, …, 10,000), we focused on SNP-miRNA pairs with eQTL effects (miR-eQTLs). The SNP-miRNA pairs with adjusted *P* value (permutation test) < 0.1 were obtained from an in-house miR-eQTL database (data not shown). Using a combination of the miR-eQTLs and clinical factors (age, sex, and number of *APOE4* alleles), a prognosis prediction model was constructed based on a Cox proportional hazard model using two thirds of the discovery cohort as defined by:

$$ h\left(t|C,I\right)={h}_0(t)\exp \left({\beta}_1\times {C}_1+\dots +{\beta}_4\times {I}_1+\dots +{\beta}_n\times {I}_m\right), $$

where *h*(*t*| *C*, *I*) is the expected hazard at time *t*, determined by a set of three covariates (*C*_1_, *C*_2_, *C*_3_ ) = (*age*, sex, number of *APOE*4 alleles) and *m* covariates (*I*_1_, *I*_2_, …, *I*_*m*_ ) whose impacts are measured by the respective coefficients (*β*_1_, *β*_2_, …, *β*_*n*_ ). Then, let *X* = {*X*_1_, …, *X*_*p*_} be the pre-selected SNP genotypes and *Y* = {*Y*_1_, …, *Y*_*q*_} be the miRNA expression values. Let *σ* = {(*x*, *y*)| *x* ∈ *X* ∧ *y* ∈ *Y*} be any SNP-miRNA pair, and let *I* = {(*x*, *y*) ∈ *σ*| miR − eQTLs} be the SNP-miRNA pairs with eQTL effects. The adjusted model was then evaluated using the remaining third of the discovery cohort. This process was repeated 3 times (3-fold cross-validation). Based on the average C-index, we determined the optimal value of *p*, the number of pre-selected SNPs for model construction. The final model was constructed using the entire discovery cohort, and the adjusted model was evaluated on the independent validation cohort.

By using the combination of miR-eQTLs and clinical factors described above, we calculated a prognostic index for each sample in the discovery cohort as defined by:

$$ \mathrm{prognostic}\ \mathrm{index}={\sum}_i^3{\beta}_i\times {C}_i+{\sum}_i^m{\beta}_{i+3}\times {I}_i. $$

We classified the samples into two groups (high and low risk) with an optimal cutoff value of the prognostic index [33]. The optimal cutoff value indicated by the minimum *P* value of the log-rank test when differences between high- and low-risk groups in the discovery cohort were compared. The optimal cutoff value was used to validate our prognosis prediction model. Kaplan-Meier curves were constructed to illustrate differences in survival without MCI-to-AD conversion. The log-rank test was used to compare the different conditions. *P* values < 0.05 were considered statistically significant. These statistical analyses were conducted using the *survival* and *survminer* packages in the statistical software R [34].

### Target gene annotation of miRNAs

The functional gene annotation of miRNAs was conducted using miRDB, which includes gene targets that are predicted to be regulated by a comprehensive set of 6709 miRNAs [35]. All gene targets have a prediction score between 0 and 100 assigned by MirTarget V3, with a higher score representing more statistical confidence in the prediction result. Only gene targets with scores of > 90 were used in our analysis.

### Network-based meta-analysis

The network-based analysis was performed with NetworkAnalyst [36] and the STRING Interactome database [37], which provides comprehensive information about interactions between proteins, including prediction and experimental interaction data. The confidence cutoff score was set to 900. The PPI network was constructed with a zero-order interaction network analysis (direct interaction only) and graphically generated using Cytoscape v3.7.2 (http://www.cytoscape.org/) [38].

### RNA-sequencing data analysis

The quality of the read sequences was assessed by using FastQC (version 0.11.7). Low-quality reads (< Q20) and trimmed reads with adapter sequences (< 50 bp) were discarded by using Cutadapt (version 1.16). The remaining clean sequenced reads were mapped to the human reference genome (GRCh37) by using STAR (2-pass option, version 2.5.2b) [39]. By using the featureCounts program [40] from the subread package (version 1.6.6), read counts for each gene were calculated to generate expression levels. The values of outliers of the read counts (i.e., the top and bottom 5% of read counts for each gene) were changed to the maximum and minimum of the remaining values, respectively. The read counts from each sample were then combined into a count file, on which differential expression analysis was performed by using edgeR [41] (version 3.18.1). Genes with a threshold CPM (counts per million reads mapped) > 1 in more than one fourth of all sequenced samples were used for further analysis. The caclNormFactorsfunction in edgeR [41] was used to obtain trimmed mean of *M* value normalization factors to account for library sizes. The dispersion was calculated by using the estimateCommonDisp and estimateTagwiseDisp functions in edgeR [41]. The exactTest function in edgeR [41] was applied to obtain genes differentially expressed between disease groups.

## Results

### Patient characteristics

The study enrolled 197 MCI patients (73 males and 124 females), followed for at least 6 months and up to 7 years (mean ± SD, 971 ± 552 days), during which time 83 (42.1%) converted to AD (i.e., 83 were classified as MCI-C). The remaining 114 patients with MCI (57.9%) were classified as MCI-NC. We divided the 197 patients with MCI into a discovery cohort of 98 individuals (41 MCI-C and 57 MCI-NC) and a validation cohort of 99 individuals (42 MCI-C and 57 MCI-NC). The patient characteristics of each cohort are summarized in Table 1.

**Table 1:** Clinical characteristics of the discovery and validation cohorts

Phenotype	Factor	All	Discovery cohort	Validation cohort
MCI-C	Number of subjects	83	41	42
	Age ± SD	75.22 ± 6.22	75.10 ± 5.64	75.33 ± 6.81
	Percentage of male (# patients)	29 (24)	41 (17)	17 (7)
	Number of *APOE4* alleles (# patients)	0 (46), 1 (29), 2 (8)	0 (25), 1 (13), 2 (3)	0 (21), 1 (16), 2 (5)
	Follow-up, mean ± SD (days)	927.18 ± 535.03	1014.07 ± 593.22	842.36 ± 462.90
MCI-NC	Number of subjects	114	57	57
	Age ± SD	75.56 ± 6.39	75.42 ± 5.75	75.70 ± 7.02
	Percentage of male (# patients)	43 (49)	44 (25)	42 (24)
	Number of *APOE4* alleles (# patients)	0 (80), 1 (32), 2 (2)	0 (40), 1 (15), 2 (2)	0 (40), 1 (17), 2 (0)
	Follow-up, mean ± SD (days)	1002.23 ± 564.49	939.70 ± 554.31	1064.75 ± 572.52
	Total subjects	197	98	99

*MCI-C* mild cognitive impairment converters (to Alzheimer’s disease), *MCI-NC* mild cognitive impairment non-converters, *SD* standard deviation

### Prognosis prediction model construction

**Fig. 1:**
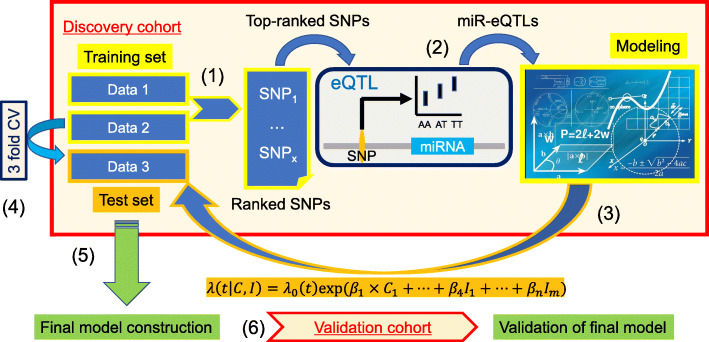
Construction workflow of our prognosis prediction model. We generated data sets of pre-selected SNPs based on the *P* values from logistic regression models. Two thirds of the entire discovery cohort was used to calculate the *P* values in each cross-validation step (1). Using the top-ranked SNPs, we focused on SNP-miRNA pairs with eQTL effects (miR-eQTLs) (2). Using a combination of miR-eQTLs and clinical factors, we constructed a prognosis prediction model based on a Cox proportional hazard model using two thirds of the discovery cohort. The adjusted model was then evaluated using the remaining one third (3). On the basis of the average C-index from the 3-fold cross-validation, we determined the optimal pre-selected SNPs for model construction (4). The final model was constructed using the entire discovery cohort (5), and the adjusted model was evaluated in the independent validation cohort (6)

**Fig. 2:**
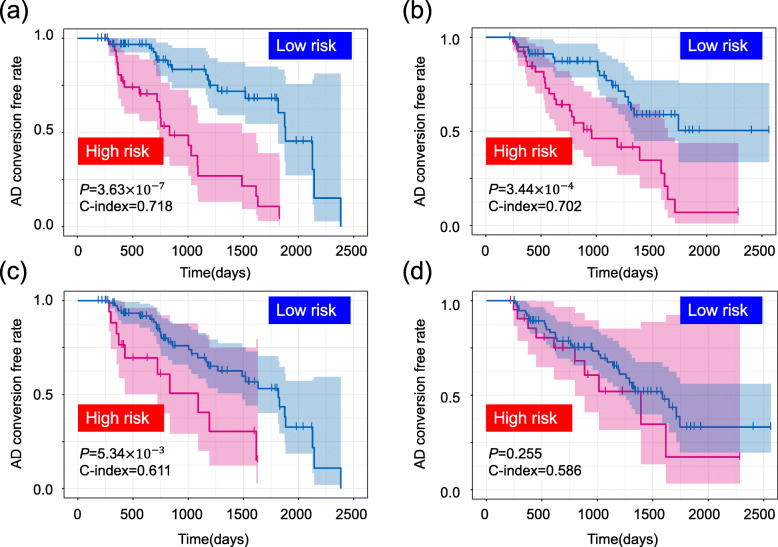
Kaplan-Meier curves of survival without conversion to AD produced by the prediction models. We calculated a prognostic index for each subject by applying the miR-eQTLs and clinical factors to our prognosis prediction model. **a** Based on the prognostic index, we divided the samples of the discovery cohort into high (red) and low (blue) risk groups. The optimal cutoff values were detected by using the minimum *P* value from the log-rank test and comparing the differences in survival without MCI-to-AD conversion as determined by Kaplan-Meier curves (optimal cutoff = 7.85, minimum *P* = 3.63 × 10^−7^). **b** The adjusted model was then evaluated on the validation cohort (log-rank test *P* = 3.44 × 10^−4^). **c**, **d** Prediction models constructed using only clinical factors (without miR-eQTLs) in the discovery cohort (**c**) and the validation cohort (**d**)

Our prognosis prediction model was based on a Cox proportional hazard method using miR-eQTLs (see details in the “Methods” section). Because evaluating all possible combinations of miR-eQTLs would be too time-consuming and computationally expensive, we generated datasets of pre-selected SNPs. The selection of the SNPs was carried out based on the *P* value of logistic regression with adjustments for three covariates: age, sex, and the number of *APOE4* alleles. Two thirds of the entire discovery cohort was used for the calculation of the *P* values. From *p* pre-selected SNPs (*p* = 100, 200, …, 10,000), we detected effective miR-eQTLs, which were obtained from an in-house miR-eQTL database (data not shown). Using a combination of the miR-eQTLs and the three clinical factors, the prognosis prediction model was constructed based on two thirds of the entire discovery cohort. The adjusted model was evaluated using the remaining third of the discovery cohort. The value of *p* that yielded highest average concordance index (C-index) across three rounds of cross-validation of the discovery cohort (Fig. 1) was selected as the optimal *p*. The final prognosis prediction model was constructed from miR-eQTLs detected from this optimal *p* (9600) pre-selected SNPs and the clinical factors using the entire discovery cohort. The adjusted model was then evaluated on the validation cohort, which was completely independent of the discovery cohort. Twenty-four miR-eQTLs were used in the final model construction, which achieved a C-index of 0.718 in the discovery cohort (Fig. 2a) and of 0.702 in the validation cohort (Fig. 2b). We also calculated a prognostic index for each subject by applying the 24 miR-eQTLs and three clinical factors to our prognosis prediction model (Table 2). We used the prognostic index to divide the discovery cohort into high- and low-risk groups. The optimal cutoff value was detected by using the minimum *P* value from the log-rank test and comparing the differences in survival without MCI-to-AD conversion as determined by Kaplan-Meier curves (optimal cutoff = 7.85, minimum *P* = 3.63 × 10^−7^, Fig. 2a). This adjusted model then successfully classified MCI patients in the validation cohort into groups with low- and high-risk of MCI-to-AD conversion (log-rank test *P* = 3.44 × 10^−4^, Fig. 2b).

**Table 2:** Potential biomarkers used in our prognosis prediction model

	Factor	*P* value^†^	Coefficient
Clinical factors	Age	NA	0.077
	Sex	NA	0.530
	*APOE4*	NA	0.602
miR-eQTLs	MIMAT0019690—rs6721935	0.092	0.181
	MIMAT0015080—rs12616298	0.089	− 0.170
	MIMAT0005582—rs12997752	0.038	− 0.141
	MIMAT0027499—rs76232851	0.093	0.042
	MIMAT0019229—rs117574479	0.032	0.152
	MIMAT0019229—rs116868325	9.69 × 10^−9^	0.058
	MIMAT0019045—rs3777118	1.10 × 10^−4^	0.034
	MIMAT0021034—rs118073044	0.037	1.078
	MIMAT0000751—rs118073044	0.078	− 3.163
	MIMAT0001630—rs118073044	0.020	1.654
	MIMAT0000086—rs117393460	0.078	0.260
	MIMAT0021033—rs72861163	0.037	0.337
	MIMAT0030997—rs9507595	0.086	− 0.123
	MIMAT0027602—rs17682567	0.072	− 0.050
	MIMAT0015080—rs117534907	0.006	− 0.032
	MIMAT0023710—rs11855092	0.014	0.156
	MIMAT0016899—rs79726130	0.054	− 0.436
	MIMAT0028122—rs79726130	0.049	0.465
	MIMAT0015080—rs35831886	1.97 × 10^−5^	1.785
	MIMAT0019229—rs35831886	0.008	− 1.688
	MIMAT0019045—rs117336092	0.031	− 0.016
	MIMAT0019229—rs117099240	0.061	0.028
	MIMAT0032029—rs149944930	0.009	0.090
	MIMAT0027487—rs2830386	0.069	0.125

^†^miR-eQTLs with adjusted *P* value < 0.1 obtained from an in-house miR-eQTL database were included

### Effectiveness of detected miR-eQTLs

To estimate the effectiveness of the 24 miR-eQTLs selected for the MCI-to-AD conversion prediction model, we compared a prediction model including the miR-eQTLs to one including only clinical factors. The prediction model excluding miR-eQTLs achieved lower C-indices (0.611 and 0.586 in the discovery and validation cohorts, respectively; Fig. 2c, d) than the model including miR-eQTLs (Fig. 2a, b). In addition, this prediction model did not successfully divide samples into high- and low-risk groups in the validation cohort (optimal cutoff = 4.39, log-rank test *P* = 0.255, Fig. 2d). These results show that the detected miR-eQTLs improve the prognosis prediction model.

**Fig. 3:**
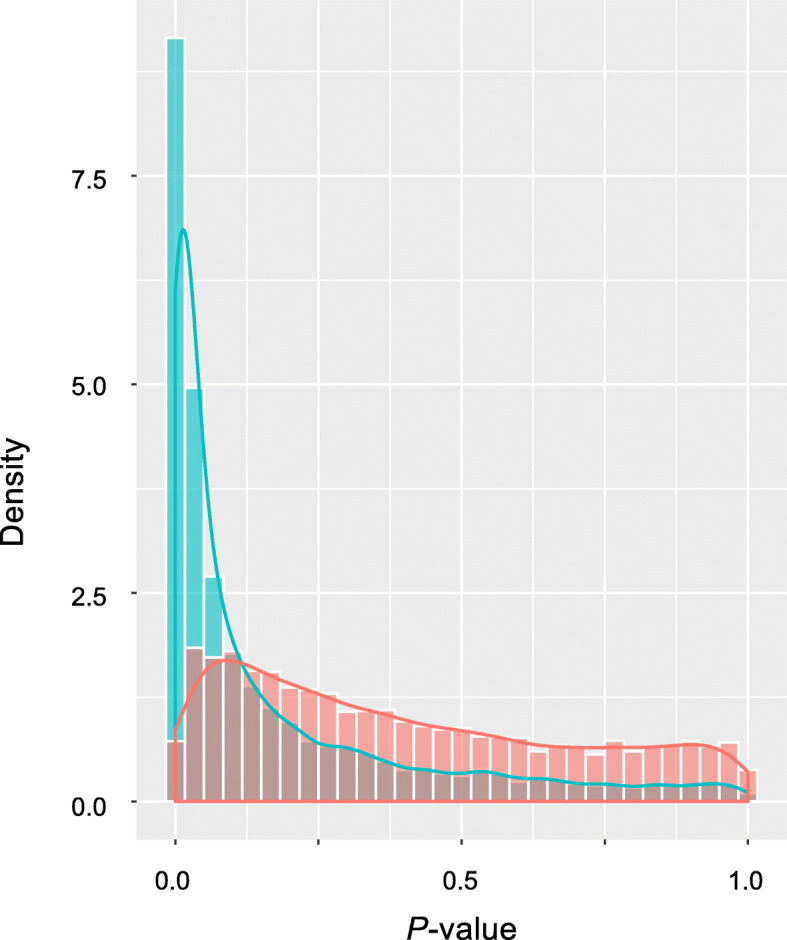
Distribution of log-ranked *P* values using a bootstrap resampling technique. We compared a prognosis model with miR-eQTLs (blue) to one without miR-eQTLs (red) using a bootstrap resampling technique. This procedure was repeated 10,000 times. The distribution of log-rank *P* values was significantly different between the prediction models with and without miR-eQTLs (*P* < 0.01, Welch’s *t* test)

To ensure the robustness and generality of our findings, we further compared prognosis models with and without miR-eQTLs with a bootstrap resampling technique. This procedure was repeated 10,000 times. The models with miR-eQTLs performed better than those with only clinical factors, and the distributions of log-rank *P* values were significantly different between the prognosis models with and without miR-eQTLs (*P* < 0.01, Welch’s *t* test) (Fig. 3).

### Functional gene annotations

**Fig. 4:**
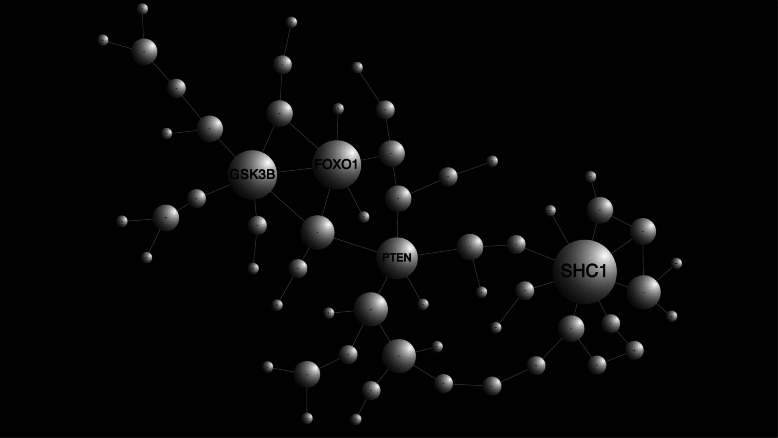
Results of the NetworkAnalyst PPI network analysis. Nodes represent genes. Node size corresponds to the number of connected edges. The gene name is displayed for nodes with ≥ 5 edges

We next examined the biological significance of our findings with miR-eQTLs by focusing on the target genes of the miRNAs. miRNAs regulate the expression of thousands of mRNAs from protein-coding genes at both the post-transcriptional and translational levels [42–44]. We predicted functional target genes of miRNAs by using the microRNA Target Prediction and Functional Study Database, miRDB [35]. Our 24 miR-eQTLs, which are composed of 20 SNPs and 18 miRNAs (Table 2 and Supplementary Table S1), were predicted to target 778 genes. We attempted to elucidate functional modules from the target genes with the occurrence of hub genes though a large-scale protein-protein interaction (PPI) network analysis. The PPI network analysis was performed by using NetworkAnalyst [36] (http://www.networkanalyst.ca) with the STRING Interactome database [37]. A PPI network generated with 2304 nodes and 3901 edges was obtained. To prune the network down to a more manageable size, we conducted a zero-order interaction network analysis and detected a refined network containing 60 nodes and 66 edges (Fig. 4). This PPI network visualization was performed with Cytoscape software [38]. Four hub genes, *GSK3B*, *PTEN*, *FOXO1*, and *SHC1*, were detected as functional modules that directly interacted with each other and with ≥ 5 other genes (Fig. 4). These four genes were regulated by the miRNAs MIMAT0032029 (hsa-miR-1249-5p), MIMAT0000086 (hsa-miR-29a-3p), MIMAT0000751 (hsa-miR-330-3p), and MIMAT0021034 (hsa-miR-5006-3p), respectively.

### Hub gene expression detected by PPI network analysis

**Fig. 5:**
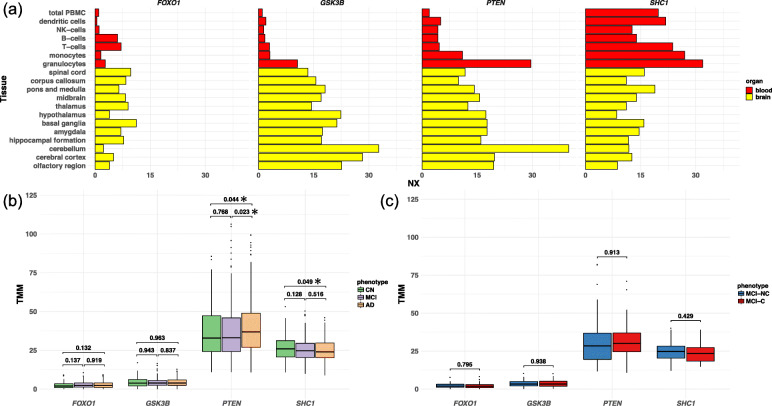
Expression of hub genes detected in the PPI network analysis. **a** The expression of all hub genes in blood cells (red) and brain tissues (yellow) were checked in the Human Protein Atlas database. An X-axis represents the resulting transcript expression values, denoted normalized expression (NX), which were calculated for each gene in every sample. **b**, **c** Gene expression was further examined using our 610 blood samples (271 from patients with AD, 248 from patients with MCI, and 91 from CNs). The difference in gene expression was examined between diseases (**b**) and between MCI-C and MCI-NC patients (*n* = 123; 48 MCI-C and 75 MCI-NC) (**c**). **b**, **c** *P* values are displayed above the boxplots. *Statistically significant differences, “exactTest” function in edgeR; TMM, trimmed mean of *M* values

Because the four hub genes (*GSK3B*, *PTEN*, *FOXO1*, and *SHC1*) were detected by PPI network analysis based on blood-based miRNA data, we checked whether these genes are actually expressed in blood cells as well as the brain by using the Human Protein Atlas database [45], which provides quantitative transcriptomics at the tissue and organ level and is publicly accessible at http://www.proteinatlas.org. Two genes (*FOXO1* and *GSK3B*) showed low levels of expression in blood, but all genes showed high levels of expression in the brain (Fig. 5a). To validate these results, we examined the expression of these genes in 610 blood samples [46] (271 ADs, 248 MCIs, and 91 cognitively normal elderly subjects [CNs]), and we investigated the differential gene expression across the disease groups. *FOXO1* and *GSK3B* showed no statistically significant difference in expression between disease groups (Fig. 5b and Supplementary Table S2). However, *PTEN* showed significantly higher gene expression in AD than in MCI (*P* = 0.023), and *SHC1* showed significantly lower expression in AD than in CN (*P* = 0.049) (Fig. 5b and Supplementary Table S2). We further investigated whether the genes were differentially expressed between MCI-C and MCI-NC patients (*n* = 123; 48 MCI-C and 75 MCI-NC), but no genes showed statistically significant differences between the two groups (Fig. 5c and Supplementary Table S3). Thus, while it might be difficult to predict the risk of AD conversion of MCI patients from these genes’ expressions, there is no doubt that these genes are associated with AD pathogenesis.

## Discussion

Early detection of individuals at high risk for MCI-to-AD conversion is important for delivering appropriate early intervention and for better managing the disease. Potential new biomarkers for this early diagnosis and prognosis have been investigated [47–50]. The role of genetic variation and blood-based miRNAs has been reviewed, with emphasis on their impact on the etiopathogenesis of sporadic AD [51, 52]. However, an integrated omics analysis of these genetic variants and miRNA expression has not, to our knowledge, been conducted in AD or other subtypes of dementia. Therefore, in this study, we investigated potential biomarkers by considering both genetic variants and miRNA expression, and we constructed a prognosis prediction model using the identified biomarkers.

We detected an optimal parameter set for the prediction model by using cross-validation of the discovery cohort. The final model was then constructed with the optimal parameters using the complete discovery cohort. The adjusted model was then evaluated on an independent validation cohort using the C-index to discriminate the accuracy of the prognosis prediction model and using survival without MCI-to-AD conversion as determined with Kaplan-Meier curves. Our final prediction model produced similar C-indices (0.718 and 0.702) in the discovery and validation cohorts and successfully classified MCI patients into two groups, low and high, in terms of risk of MCI-to-AD conversion (log-rank test *P* = 3.63 × 10^−7^ and 3.44 × 10^−4^). These results demonstrate that miR-eQTLs could be efficient biomarkers for the early detection of individuals at high risk of MCI-to-AD conversion, although replication studies using a larger number of samples are necessary. Bootstrap resampling indicated that prediction models with miR-eQTLs had superior performance to those with clinical factors only (without miR-eQTLs). These results provide evidence that our findings can be efficiently applied to early prediction of MCI-to-AD conversion, which is expected to contribute to practical clinical use in MCI-to-AD conversion in the near future.

Our findings represent SNP-miRNA pairs with eQTL effects. Each SNP was associated with variation of miRNA expression levels. These miRNAs act as post-transcriptional regulators of their mRNA targets through mRNA degradation and/or translational repression. Therefore, annotation of the mRNA gene targets can lead to functional characterization of our findings. The functional gene annotation of miRNAs was conducted using miRDB [35], and four functionally important modules (i.e., hub genes, *GSK3B*, *PTEN*, *FOXO1*, and *SHC1*) were detected from large-scale PPI network analysis. *FOXO1* is a member of the evolutionarily conserved FOXO family of transcription factors. Although only *FOXO3* has been analyzed in AD models so far, the gene has been reported to induce cell death in response to amyloid beta plaques and to influence the function of the peripheral and central nervous systems [53]. *GSK3B* has been identified as a tau protein kinase I, which phosphorylates tau at several sites [54]. This abnormally hyperphosphorylated tau is generally observed in the brains of patients with AD [55]. Although *PTEN* is best known as a tumor suppressor gene, this gene has also been reported to be associated with other diseases, including diabetes and AD [56]. Liang et al. reported that *SHC1* might play a key role in the progression of AD [57]. Also, Zheng et al. recently ranked 500 genes according to their potential association with AD risk, and *SHC1* was in the top 20 [58]. In this study, we further investigated the expression of these hub genes in CN, MCI, and AD blood samples. Whereas *FOXO1* and *GSK3B* showed low levels of expression in all of the samples, *PTEN* and *SHC1* showed a significant difference in gene expression in the blood between diseases. These results suggest that these identified hub genes are associated with the pathogenesis of AD.

We have proposed an MCI-to-AD conversion prediction model based on a Cox proportional hazard method using SNP-miRNA pairs with eQTL effects. Our proposed model may enable early detection of patients at high risk of MCI-to-AD conversion. However, further refinement of this model, using a larger number of samples, will be required before it can be used in health care. Omics analyses of genetic variations, such as SNPs, insertions and deletions (indels), and gene expression, will play an important role in the further improvement of this prognosis prediction model.

### Limitations

There are limitations of the current analyses. As it is too difficult to collect many MCI converters and MCI non-converters, our prediction model is constructed using Japanese MCI patients with limited sample size. In the future, we will perform further investigations with larger sample size and will further validate the effectiveness of this classifier.

## Conclusions

Our final prediction model successfully classified MCI patients into low and high risk of MCI-to-AD conversion and achieved a high concordance index on an independent test set. Important hub genes associated with AD pathogenesis were also identified in a network-based meta-analysis of miR-eQTL target genes. Accurate prediction of MCI-to-AD conversion would enable earlier intervention for MCI patients at high risk, potentially reducing conversion to AD.

## Supplementary Information

**Additional file 1:** **Supplementary Table S1.** SNP genotypes included in miR-eQTLs as potential biomarkers.

**Additional file 2:** **Supplementary Table S2.** Gene expression in 610 blood samples.

**Additional file 3:** **Supplementary Table S3.** Gene expression in 123 MCI blood samples.

## Data Availability

All microarray data (2562 miRNAs) and clinical information from this study are publicly available through the Gene Expression Omnibus (GEO) database at the National Center for Biotechnology Information (NCBI) and accessible through GEO series accession number GSE150693 at http://www.ncbi.nlm.nih.gov/projects/geo/. The other datasets used or analyzed in the current study are available from the corresponding author on reasonable request.
