# Supplementary material for: Prognosis prediction model for conversion from mild cognitive impairment to Alzheimer’s disease created by integrative analysis of multi-omics data
Source: Alzheimers Res Ther. 2020 Nov 10;12:145. doi: 10.1186/s13195-020-00716-0 (PMC7656734; doi:10.1186/s13195-020-00716-0)
Supplement: Supplementary file 1 — Additional file 1: Supplementary Table S1. SNP genotypes included in miR-eQTLs as potential biomarkers. [file 13195_2020_716_MOESM1_ESM.docx]

**Supplementary Table S1. SNP genotypes included in miR-eQTLs as potential biomarkers**

| SNP |  | MCI-C | | | MCI-NC | | | OR (95% CI) | *P*-value |
| --- | --- | --- | --- | --- | --- | --- | --- | --- | --- |
|  | Allele 1/2 | 11 | 12 | 22 | 11 | 12 | 22 |  |  |
| rs6721935 | T/C | 1 | 10 | 30 | 0 | 9 | 48 | 2.26 (0.84 to 6.05) | 0.11 |
| rs12616298 | G/A | 0 | 1 | 40 | 0 | 6 | 51 | 0.19 (0.022 to 1.69) | 0.14 |
| rs12997752 | C/T | 0 | 1 | 40 | 0 | 7 | 50 | 0.19 (0.022 to 1.64) | 0.13 |
| rs76232851 | A/C | 0 | 3 | 38 | 0 | 12 | 45 | 0.29 (0.073 to 1.14) | 0.077 |
| rs117574479 | G/A | 0 | 4 | 37 | 0 | 1 | 56 | 6.59 (0.68 to 63.93) | 0.10 |
| rs116868325 | C/A | 0 | 4 | 37 | 0 | 1 | 56 | 6.26 (0.66 to 58.94) | 0.11 |
| rs3777118 | G/A | 0 | 5 | 36 | 0 | 2 | 55 | 3.82 (0.68 to 21.36) | 0.13 |
| rs118073044 | A/G | 1 | 4 | 36 | 0 | 2 | 55 | 3.82 (0.73 to 19.87) | 0.11 |
| rs117393460 | T/C | 0 | 8 | 33 | 0 | 3 | 54 | 4.26 (1.04 to 17.41) | 0.043 |
| rs72861163 | T/C | 0 | 6 | 35 | 0 | 2 | 55 | 5.78 (1.07 to 31.23) | 0.041 |
| rs9507595 | C/T | 4 | 27 | 10 | 17 | 27 | 13 | 0.61 (0.33 to 1.14) | 0.12 |
| rs17682567 | C/T | 0 | 1 | 40 | 0 | 7 | 50 | 0.20 (0.023 to 1.69) | 0.14 |
| rs117534907 | C/T | 1 | 3 | 37 | 0 | 1 | 56 | 5.25 (0.62 to 44.08) | 0.13 |
| rs11855092 | A/G | 0 | 6 | 35 | 0 | 2 | 55 | 5.83 (1.08 to 31.60) | 0.041 |
| rs79726130 | G/A | 0 | 4 | 37 | 0 | 1 | 56 | 8.11 (0.82 to 79.74) | 0.073 |
| rs35831886 | C/T | 1 | 4 | 36 | 0 | 1 | 56 | 7.76 (0.89 to 67.93) | 0.064 |
| rs117336092 | G/T | 0 | 6 | 35 | 0 | 3 | 54 | 3.11 (0.71 to 13.75) | 0.13 |
| rs117099240 | A/G | 0 | 6 | 35 | 0 | 2 | 55 | 5.20 (0.95 to 28.49) | 0.058 |
| rs149944930 | A/G | 0 | 5 | 36 | 0 | 2 | 55 | 4.17 (0.75 to 23.10) | 0.10 |
| rs2830386 | A/G | 11 | 19 | 11 | 5 | 34 | 18 | 1.68 (0.89 to 3.15) | 0.11 |

OR; odds ratio, CI; confidence interval.

The *P*-values were calculated by logistic regression with adjustments for three covariates: age, sex, and number of *APOE4* alleles. All SNPs were mapped to the human reference genome (GRCh37).
